# Beyond cardiovascular protection: a conceptual and translational review of the hepato-specific mechanisms of statins in metabolic dysfunction-associated steatotic liver disease (MASLD)

**DOI:** 10.3389/fphar.2026.1884657

**Published:** 2026-07-23

**Authors:** Carolina Jiménez-González, Paula Iruzubieta, Marta Alonso-Peña, Lorena Cayón, Javier Crespo

**Affiliations:** 1 Gastroenterology and Hepatology Department, Clinical and Translational Research in Digestive Diseases, Valdecilla Research Institute (IDIVAL), Marqués de Valdecilla University Hospital, Santander, Spain; 2 Departamento de Anatomía y Biología Celular, Universidad de Cantabria, Santander, Spain; 3 Cantabria Cohort, Valdecilla Research Institute (IDIVAL), Marqués de Valdecilla University Hospita, Santander, Spain; 4 School of Medicine, University of Cantabria, Santander, Spain; 5 Medicine AI, Madrid, Spain

**Keywords:** isoprenoids, KLF2, MASH, MASLD, mevalonate pathway, NLRP 3, statins, YAP/TAZ

## Abstract

Metabolic dysfunction-associated steatotic liver disease (MASLD) is the most prevalent chronic liver disease worldwide, and cardiovascular disease remains the leading cause of death in this population. Statins are therefore a cornerstone of therapy in MASLD because of their antiatherosclerotic efficacy. Less attention has been paid to the possibility that part of the hepatic benefit observed in MASLD cohorts—including lower aminotransferase levels, slower fibrosis progression, reduced decompensation and lower hepatocellular carcinoma incidence—may reflect direct hepatic effects beyond LDL reduction. Although the evidence is predominantly observational, the direction and magnitude of the association have remained broadly consistent across independent cohorts. Recent studies have strengthened this signal, including a *post hoc* analysis of the PROSPER trial showing attenuation of excess mortality in individuals with elevated FIB-4, and a large Veterans Affairs cohort demonstrating a dose-dependent association between cumulative statin exposure and lower primary liver cancer risk in MASLD. This review integrates the preclinical, observational and indirect randomized evidence supporting the biological plausibility of liver-directed effects of statins. Mechanistically, the argument centres on inhibition of the mevalonate pathway as a molecular hub linking lipotoxicity, NLRP3-dependent inflammation, fibrogenesis and carcinogenesis through impaired prenylation of small GTPases. A fifth axis, intrahepatic hemodynamics mediated through KLF2/eNOS signaling, is supported by randomized evidence in compensated cirrhosis. In contrast, the LIVERHOPE-EFFICACY trial showed no benefit in decompensated cirrhosis, helping define the therapeutic window. Within the limitations of the available evidence, we propose a conceptual reframing: in MASLD, statins may act not only as cardiovascular drugs, but also as agents with potentially relevant hepatic effects mediated through a shared molecular substrate.

## 1 Introduction

The MASLD designation, adopted in 2023 through a multisociety Delphi consensus, replaced NAFLD with intent that extended beyond terminology (Rinella et al., 2023). Positive inclusion by cardiometabolic criteria articulated a paradigm shift: hepatic steatosis was to be understood not as a diagnosis of exclusion but as the hepatic manifestation of a systemic disorder, in which metabolic dysfunction precedes and sustains liver injury (Tacke et al., 2024). Under this lens, the fact that cardiovascular disease—rather than liver disease—remains the leading cause of death in MASLD is not surprising (Targher et al., 2020): it is the pathogenic logic itself that predicts this outcome. Recent reviews have further emphasized the bidirectional relationship between MASLD and cardiovascular disease, identifying endothelial dysfunction as a common pathophysiological denominator linking hepatic injury, atherosclerosis and adverse cardiovascular outcomes (Ktenopoulos et al., 2024).

This rationale supports the central role of statins in MASLD, made explicit in the 2024 EASL-EASD-EASO guideline (Tacke et al., 2024). The dominant interpretive framework—statin as a cardiovascular drug that the liver tolerates safely—is not in dispute. What merits closer examination is whether this framework captures the totality of the observed effect. Over the past decade, several cohorts have accumulated observations that are difficult to attribute to LDL reduction alone. The Korean nationwide study of 516,575 individuals by Yun and colleagues reported associations between statin use and a 36% reduction in major hepatic events, 48% in HCC and 42% in decompensated cirrhosis in MASLD/MetALD (Yun et al., 2025). The multicentre VCTE-Prognosis study of 7,988 patients with longitudinal elastography documented attenuation of liver stiffness progression in statin users, alongside lower mortality and fewer hepatic events (Zhou et al., 2024). The meta-analysis by Zeng and colleagues, comprising 23 matched studies, placed the association between statin use and reduced HCC incidence at HR 0.52, with an apparently larger magnitude for lipophilic statins (Zeng et al., 2023).

Two publications from 2025 to 2026 have added qualitatively different information. The *post hoc* analysis of PROSPER in patients with elevated FIB-4 represents, to date, the randomized evidence closest to the question, although—as we shall discuss—its interpretation requires caution (de Jong et al., 2025). The VA cohort of Karachaliou and colleagues provides a robust dose-response analysis with stratification by age, sex, insulin resistance and cirrhosis status (Karachaliou et al., 2026).

The consistency of the effect direction, its relative magnitude and its reproducibility across cohorts admit two readings. The first is that the association reflects a persistent systematic artefact: confounding by indication, healthy user effect, immortal time bias. The second is that we are facing a real signal that the prevailing interpretive framework fails to capture. This review does not aim to decide between the two. We argue, rather, that the convergence between the observed clinical pattern and inhibition of the mevalonate pathway—with its consequences for small GTPase prenylation—makes the second reading worth exploring without dismissing the first. Unlike previous reviews that have addressed the pleiotropic effects of statins as discrete observations, this review proposes a unifying mechanistic framework in which inhibition of the mevalonate pathway acts as the common molecular substrate linking lipotoxicity, inflammation, fibrogenesis, carcinogenesis and intrahepatic vascular dysfunction. Integrating these pathways with the emerging clinical evidence in MASLD allows us to outline a conceptual model that may account for the reproducible hepatic signal observed across independent cohorts. The framework we propose does not compete with cardiovascular protection: it shares its molecular substrate.

## 2 The observational paradox and its methodological limitations

Before exploring mechanism, it is necessary to delimit what the cohorts observe and, more importantly, what biases might account for the observation. The clinical signal motivating this review is real, but most of the available evidence is observational and therefore vulnerable to several specific sources of error.

Confounding by indication comes first. Patients who receive statins differ systematically from those who do not: they often have a higher burden of cardiovascular comorbidity, but also better overall adherence, greater use of healthcare resources and concurrent prescribing patterns (aspirin, metformin, antihypertensives) that may themselves contribute to outcomes (Shrank et al., 2011; Patrick et al., 2011; Brookhart et al., 2007). The healthy user effect is notoriously difficult to neutralize through conventional statistical adjustment, particularly when exposure is defined by chronic prescription of a preventive drug.

Immortal time bias has been a recurrent companion of this literature. In designs that classify statin exposure on the basis of future or cumulative prescriptions, patients must survive long enough to enter the exposed category, artificially inflating the apparent benefit (Levesque et al., 2010; Abrahami et al., 2021). More careful studies address this with time-varying analyses or emulated target trial designs (Xu et al., 2024), but practice is heterogeneous and meta-analyses inherit that heterogeneity.

A third issue is specific to the oncological domain: the long latency between exposure and outcome. HCC has a protracted induction period, which multiplies the opportunities for residual confounding and demands analyses based on cumulative exposure rather than binary indicators (Lee et al., 2009).

A fourth limitation is unmeasured residual confounding. Variables such as exercise adherence, dietary quality, undisclosed alcohol use or hepatoprotective supplementation are not captured in administrative databases, yet may be differentially distributed between users and non-users.

The fifth, and potentially the most significant, limitation is the lack of randomized trials with primary histological endpoints in pre-cirrhotic MASLD. The randomized piece closest to the question is the *post hoc* analysis of PROSPER, and it deserves specific discussion because it carries genuine value alongside a clear interpretive ceiling. PROSPER randomized 5,804 participants aged over 70 to pravastatin 40 mg/day or placebo, with cardiovascular primary endpoints and a median follow-up of 3.2 years. In the FIB-4–stratified analysis, patients with FIB-4 ≥2.67 showed significant excess all-cause mortality in the placebo arm, which was attenuated in the pravastatin arm (de Jong et al., 2025). The finding is meaningful: it shows that the direction of effect seen in cohorts is consistent with what a randomized design detects when stratified by a non-invasive marker of hepatic severity. It should, however, be read precisely. PROSPER was not designed for MASLD, did not employ histological or hepatic primary endpoints, and FIB-4 correlates imperfectly with advanced MASLD in elderly populations. Moreover, age-adjusted FIB-4 thresholds were not applied, which may have limited the specificity of fibrosis classification in this elderly population. What it offers is indirect randomized evidence—hypothesis-generating rather than causally definitive.

Operational heterogeneity across studies must finally be acknowledged. Definitions of MASLD (FLI, ICD-10, biopsy, elastography), of statin exposure (any prescription, continued coverage, cumulative dose), of induction time and of follow-up duration differ between cohorts, complicating quantitative synthesis.

There remain, nonetheless, reasons to pursue mechanism rather than dismiss the signal. The relative magnitude of the observed effect exceeds what one would expect from conventional confounding, though it does not exclude it. Reproducibility across cohorts of very different design and population reduces the likelihood of a single systematic artefact. And the biological plausibility, examined below, is traceable to specific molecular mechanisms. It is the convergence of these three dimensions—not any one alone—that justifies the remainder of this paper.

## 3 The liver as a privileged pharmacokinetic target

Any discussion of hepato-specific effects should begin with a basic pharmacological observation that is rarely emphasized in the MASLD literature: the hepatocyte is the cell type where the molecule concentrates. All statins are hepato-selective by virtue of first-pass uptake (Schachter, 2005). Lipophilic statins (atorvastatin, simvastatin, lovastatin, fluvastatin, pitavastatin) enter the hepatocyte by passive diffusion; hydrophilic statins (pravastatin, rosuvastatin) require active uptake through OATP1B1, the transporter encoded by SLCO1B1 and expressed specifically on the sinusoidal membrane of the hepatocyte (Commins et al., 2026).

The pharmacokinetic consequence is that hepatic statin exposure substantially exceeds systemic exposure, often by one order of magnitude or more, depending on the specific agent and on hepatic transporter activity (Schachter, 2005; Commins et al., 2026; Ahmadi et al., 2020). This hepato-selectivity has a dual implication: it explains why systemic inhibition of HMG-CoA reductase at therapeutic doses does not produce massive extrahepatic toxicity, and it predicts that any pleiotropic effect mediated by mevalonate pathway inhibition should be quantitatively more intense in the liver than in any other organ (Ahmadi et al., 2020; Ahmadi et al., 2017). What is a modest pleiotropic effect in vascular endothelium may be biologically substantial in hepatocytes, Kupffer cells, hepatic stellate cells and sinusoidal endothelium. From a pharmacokinetic standpoint, hepato-specific effects require no additional explanation: they are the expected consequence of drug distribution (Palatini and De Martin, 2016).

The lipophilic/hydrophilic distinction is relevant here. Both classes inhibit HMG-CoA reductase with comparable efficacy on LDL at equipotent doses, but their intrahepatic distribution differs. Lipophilic statins also penetrate non-parenchymal cells with limited OATP1B1 expression (Kupffer cells, HSCs, sinusoidal endothelium); hydrophilic statins remain more confined to the hepatocyte (Climent et al., 2021; Ahsan et al., 2020). This difference provides a plausible biological basis for differential hepatic effects, although its clinical significance remains uncertain. At present, no randomized or prospective comparative data support the superiority of lipophilic over hydrophilic statins for hepatic outcomes.

## 4 The mevalonate pathway as a molecular hub in the steatotic liver

**FIGURE 1:**
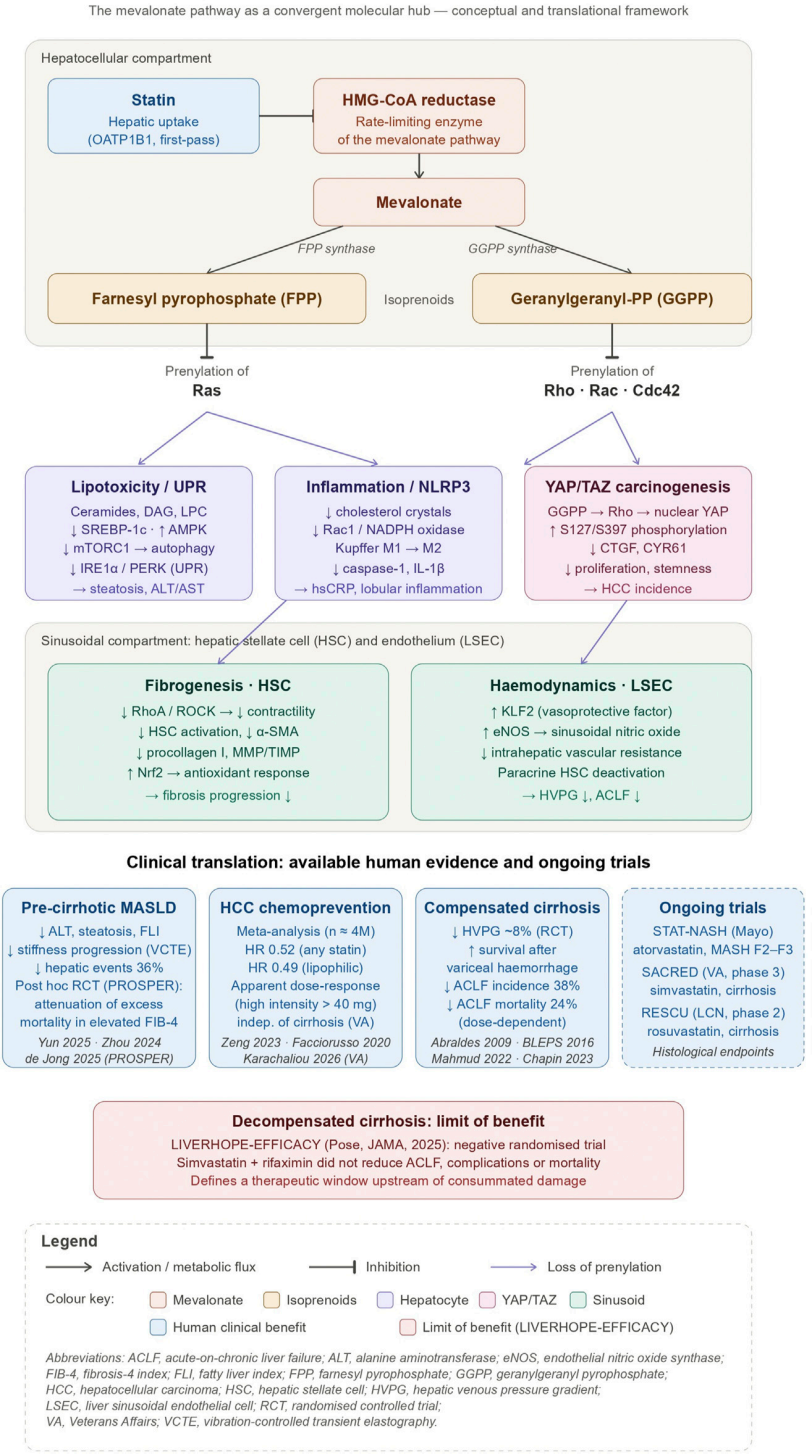
Statins in MASLD: hepato-specific mechanisms. Integrated mechanistic diagram articulating the hepato-specific hypothesis of the manuscript. The top band represents the pharmacological input: the statin, selectively taken up by hepatocytes through OATP1B1, inhibits HMG-CoA reductase and reduces flux through the mevalonate pathway. The bifurcation into FPP and GGPP governs the prenylation of small GTPases (Ras via FPP; Rho, Rac and Cdc42 via GGPP), the upstream molecular event organizing the five pathogenic axes shown in the subsequent bands. Three axes are hepatocellular (lipotoxicity/UPR; NLRP3 inflammation; YAP/TAZ-driven carcinogenesis) and two sinusoidal (fibrogenesis via HSC; hemodynamics via LSEC through KLF2/eNOS). The lower band presents the human clinical translation across four panels: pre-cirrhotic MASLD, HCC chemoprevention, compensated cirrhosis and ongoing trials. The red band at the bottom delimits the therapeutic window: decompensated cirrhosis, where LIVERHOPE-EFFICACY demonstrated no benefit. Abbreviations are shown within the figure itself.

**FIGURE 2:**
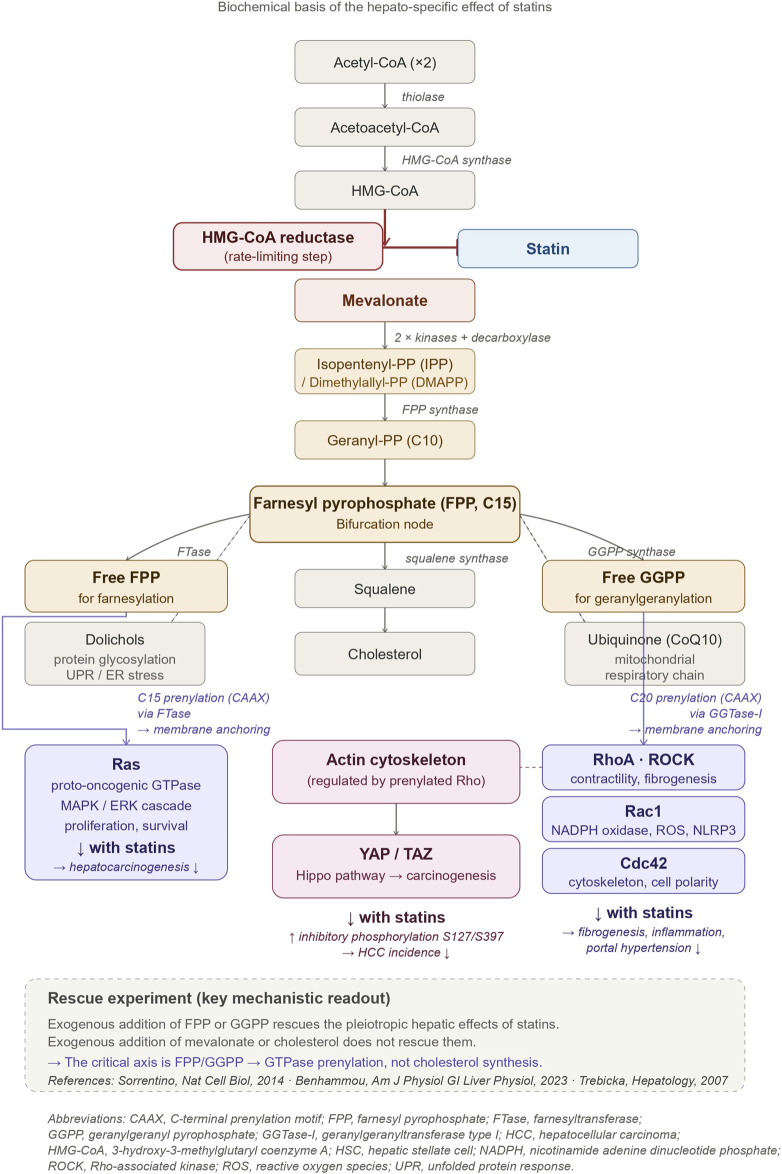
Molecular detail: mevalonate pathway and prenylation of GTPases. Expanded panel showing the complete biochemical cascade from acetyl-CoA to the terminal products (cholesterol, dolichols, ubiquinone) and, laterally, the two critical isoprenoid branches. Free FPP, substrate for farnesyltransferase (FTase), mediates the prenylation of Ras (C15). Free GGPP, substrate for geranylgeranyltransferase type I (GGTase-I), mediates the prenylation of Rho, Rac and Cdc42 (C20). The actin cytoskeleton, regulated by prenylated Rho proteins, connects with the Hippo pathway and the nuclear translocation of YAP/TAZ. The lower box summarises the rescue experiment that anchors the mechanistic model: exogenous addition of FPP or GGPP restores many of the pleiotropic effects of statins, supporting a central role for isoprenoid-dependent signalling. Cholesterol supplementation does not reproduce these effects. Abbreviations are shown within the figure itself.

Inhibition of HMG-CoA reductase by statins does not merely reduce cholesterol synthesis. It simultaneously reduces the availability of the intermediate isoprenoids derived from the mevalonate pathway. Farnesyl pyrophosphate (FPP) and geranylgeranyl pyrophosphate (GGPP) are the lipid donors for the post-translational prenylation of small GTPases of the Ras family (Ras, Rho, Rac, Cdc42), a covalent modification indispensable for their membrane anchoring and therefore for their function as signaling switches (Oesterle et al., 2017; McTaggart, 2006), as described in Figures 1, 2. Inhibition of prenylation traps these GTPases in an inactive cytoplasmic conformation, profoundly modifying downstream signal transduction.

Four downstream branches of the mevalonate pathway are relevant in MASLD. Farnesylation of Ras governs proliferative and antiapoptotic signaling. Geranylgeranylation of RhoA, Rac1 and Cdc42 regulates the actin cytoskeleton, cellular contractility (in HSCs), NADPH oxidase activation and nuclear translocation of YAP/TAZ. Dolichol synthesis modulates protein glycosylation and the UPR; ubiquinone synthesis underpins mitochondrial respiratory chain function. These branches do not operate in isolation: the four major pathogenic axes of MASLD progression—lipotoxicity/UPR, NLRP3-dependent inflammation, fibrogenesis/HSC and YAP/TAZ-driven carcinogenesis—depend, fully or partly, on signaling tied to prenylated small GTPases.

Multiple experimental studies have shown that restoring isoprenoid intermediates, particularly GGPP, reverses many of the pleiotropic cellular effects of statins (Sorrentino et al., 2014; Benhammou et al., 2023). These observations support the view that disruption of protein prenylation, rather than cholesterol depletion itself, constitutes a central mechanistic mediator of statin action. The relative contribution of upstream mevalonate supplementation appears to be context-dependent and varies with cell type and experimental design. The critical component of the hepato-specific effect therefore lies not in cholesterol synthesis but in the availability of intermediate isoprenoids.

## 5 Four hepato-specific pathogenic axes potentially modulated by statins

### 5.1 Lipotoxicity and endoplasmic reticulum stress

Accumulation of saturated fatty acids—palmitate in particular—in the steatotic hepatocyte triggers a lipotoxic cascade that includes ceramide synthesis, lysophosphatidylcholine and diacylglycerol generation, activation of stress kinases (MLK3, JNK), engagement of the UPR (IRE1α, PERK, ATF6) and, ultimately, apoptosis (Hirsova et al., 2016; Kakazu et al., 2016). Activation of IRE1α-XBP1 links endoplasmic reticulum stress to lipogenesis through SREBP-1c, perpetuating steatosis.

Statins may influence this axis through two converging mechanisms. Inhibition of hepatic cholesterol synthesis and the resulting feedback on SREBP-2 reorganise hepatic lipid metabolism and reduce the availability of potentially lipotoxic substrates. Inhibition of mTORC1 and activation of AMPK, observed with statins in experimental models—particularly with lipophilic agents—promote autophagy and, specifically, lipophagy. Zhang and colleagues showed in murine models that combined lovastatin and ezetimibe activate the SREBP-2 → PNPLA8 → autophagy axis, reducing hepatic triglycerides (Kim et al., 2016).

The observational human correlate is consistent, as shown in Table 1. Statins are associated with reductions in ALT and AST in MASLD cohorts, without signal of significant hepatotoxicity in 12-month follow-ups, including the recent Malaysian cohort of 104 patients (Yun et al., 2025; Ayada et al., 2023; Wong et al., 2025). There is, however, no randomized evidence with primary histological endpoints quantifying the effect on lipotoxicity in pre-cirrhotic MASLD. The ongoing STAT-NASH trial (NCT04679376) is designed precisely to fill that gap by evaluating atorvastatin versus placebo in statin-naïve patients with MASH and F2–F3 fibrosis. Histological improvement is the primary endpoint, making it the first trial specifically designed to determine whether statin therapy can directly modify liver histology in pre-cirrhotic disease.

**TABLE 1:** Hepato-specific mechanisms of statins in MASLD: level of evidence and clinical translation The table summarises the five mechanistic axes discussed in the manuscript. The final column classifies the level of inference of the available evidence—experimental, observational, indirect RCT or direct clinical RCT—to avoid confusion between distinct epistemological planes.

Pathogenic axis	Basic molecular mechanism	Expected clinical translation	Available human evidence	Level of inference
Lipotoxicity/Endoplasmic reticulum stress	Feedback inhibition of SREBP-1c; AMPK activation; mTORC1 inhibition; induction of autophagy/lipophagy; reduction of ceramides and LPC; modulation of UPR (IRE1α, PERK)	Association with reduced hepatic steatosis, aminotransferases, NAS; improved hepatocellular metabolic milieu	Consistent reductions in ALT/AST and FLI in cohorts (Yun et al., 2025; Ayada et al., 2023; Wong et al., 2025)	Experimental + observational. STAT-NASH (ongoing, phase 2) will provide randomized evidence with histological endpoint
Hepatic inflammation and innate immunity (NLRP3/cholesterol crystals)	Reduction of intrahepatic cholesterol crystals (NLRP3 activator documented in human biopsies (Ioannou et al., 2013; Ioannou et al., 2017); Rac1 inhibition → NADPH oxidase (NOX2) → ROS; M1→M2 Kupffer modulation	Association with reduced lobular inflammation; potential reduction in IL-1β and IL-18	No specific human RCT. Indirect evidence through reduction of hsCRP and ALT	Experimental, with partially conflicting evidence on NLRP3 as a sole mediator (Mridha et al., 2017; Ioannou et al., 2023). No direct clinical validation
Fibrogenesis (HSC and LSEC)	Inhibition of RhoA geranylgeranylation → ROCK blockade; induction of KLF2 in LSECs → restoration of eNOS/NO → paracrine HSC deactivation; Nrf2-mediated antioxidant activation	Association with attenuated fibrosis progression (VCTE, FIB-4); potential cACLD regression; attenuation of excess mortality in elevated FIB-4	Robust observational (Zhou et al., 2024; Ayada et al., 2023; Schreiner et al., 2024; Pustjens et al., 2026). Post hoc RCT in PROSPER (de Jong et al., 2025)	Robust experimental + consistent observational + indirect RCT. SACRED and RESCU (ongoing) will provide direct RCT.
Hepatic carcinogenesis (YAP/TAZ axis)	Inhibition of GGPP → reduced Rho GTPase prenylation → reduced YAP/TAZ activation via Hippo; increased inhibitory YAP S127/S397 phosphorylation; modulation of HCC risk gene signature	Association with reduced primary and recurrent HCC incidence; apparently consistent dose-response	Meta-analyses HR 0.52 [7]; lipophilic HR 0.49 [49] VA cohort with dose-response in MASLD (Karachaliou et al., 2026). NHIS-Senior ≥60 years (Kang et al., 2026)	Experimental + observational with dose-response. No specific RCT. Subject to immortal time bias and concomitant prescription confounding
Intrahepatic hemodynamics and the decompensation–ACLF spectrum	KLF2 activation in LSECs; restoration of eNOS and sinusoidal NO production; reduction of intrahepatic vascular resistance; inhibition of RhoA/ROCK	HVPG reduction; improved post-variceal bleeding survival; lower ACLF incidence and mortality in compensated cirrhosis. No benefit in decompensated cirrhosis	Positive RCTs in compensated cirrhosis (Abraldes et al., 2009; Abraldes et al., 2016) VA observational (Mahmud et al., 2022; Chapin et al., 2023). NEGATIVE RCT in decompensated (Pose et al., 2025)	Direct clinical RCT (compensated, phase 2). Negative RCT (decompensated). Highest level of evidence in the review

Abbreviations: ACLF, acute-on-chronic liver failure; ALT, alanine aminotransferase; AMPK, AMP-activated protein kinase; AST, aspartate aminotransferase; cACLD, compensated advanced chronic liver disease; cDDD, cumulative defined daily dose; eNOS, endothelial nitric oxide synthase; FIB-4, fibrosis-4 index; FLI, fatty liver index; GGPP, geranylgeranyl pyrophosphate; HCC, hepatocellular carcinoma; HSC, hepatic stellate cell; HVPG, hepatic venous pressure gradient; KLF2, Krüppel-like factor 2; LPC, lysophosphatidylcholine; LSEC, liver sinusoidal endothelial cell; NAS, NAFLD, activity score; NO, nitric oxide; PLC, primary liver cancer; RCT, randomized controlled trial; SREBP, sterol regulatory element-binding protein; UPR, unfolded protein response; VA, veterans affairs; VCTE, vibration-controlled transient elastography.

### 5.2 Hepatic inflammation and innate immunity: the NLRP3 axis and its nuances

Hepatic inflammation in MASH has a well-characterized molecular coordinator: the NLRP3 inflammasome, expressed primarily in Kupffer cells and infiltrating macrophages. Its activation assembles the NLRP3-ASC-caspase-1 complex, cleaves pro-IL-1β to mature IL-1β and drives pyroptosis. Ioannou and colleagues demonstrated, in human MASH biopsies and in murine models, that intrahepatic cholesterol crystals—absent in simple steatosis—specifically activate NLRP3 within the crown-like structures surrounding steatotic hepatocytes (Ioannou et al., 2013; Ioannou et al., 2017). Mridha and colleagues extended this evidence by showing that pharmacological blockade of NLRP3 with MCC950 reduces inflammation, ALT and fibrosis in foz/foz and MCD models (Mridha et al., 2017).

Statins target two points along this axis. By reducing intracellular hepatic cholesterol and modulating its trafficking, they diminish cholesterol crystal formation, the upstream molecular trigger. Inhibition of Rac1 prenylation additionally reduces NOX2 NADPH oxidase assembly, an additional priming signal. Inia JA and colleagues confirmed this in the APOE*3-Leiden NASH model: atorvastatin reduced the histologically quantified burden of intrahepatic cholesterol crystal formation by 78%, decreased hepatic IL-1β expression by 61%, and was associated with reductions in lobular inflammatory lesions and histological fibrosis area of 80% and 92%, respectively (Inia et al., 2023).

The evidence is not unanimous. A later study by the Ioannou group—the same that originally characterized the role of cholesterol crystals—showed that neither genetic deletion of NLRP3 nor its pharmacological inhibition with MCC950 attenuates liver injury in two murine NASH models induced by cholesterol-rich diets (Ioannou et al., 2023). That observation qualifies, without invalidating, the centrality of NLRP3: it likely acts as one of several convergent effectors rather than as a sole coordinator. Hepatic inflammation in MASLD is orchestrated by a broader network that includes TLR-dependent signaling, NF-κB activation, cytokine-mediated crosstalk and multiple innate immune pathways capable of sustaining liver injury even when a single node is inhibited. In this context, NLRP3 may be better viewed as one component of a partially redundant inflammatory system rather than as an exclusive driver of disease progression. For the argument advanced here, this complexity does not weaken the rationale for statin effects; what it precludes is presenting NLRP3 blockade as a sufficient mechanism. Early intervention on cholesterol crystals—before the inflammatory network is fully established—remains a reasonable hypothesis compatible with the full body of data.

In humans with MASH, no clinical trials have been specifically designed to quantify the effect of statins on inflammasome activity. The indirect evidence—reduction in ALT, hsCRP and IL-6, and modulation towards an M2 polarization in animal models (Schierwagen et al., 2016)—supports the direction of effect but not its magnitude.

### 5.3 Fibrogenesis: the Rho/ROCK axis and the induction of KLF2

Hepatic fibrogenesis is driven principally by activated hepatic stellate cells (HSCs), which transdifferentiate from a quiescent retinoid-storing phenotype to a myofibroblast-like phenotype that produces type I collagen, expresses α-SMA and is contractile. This activation depends critically on RhoA-ROCK signaling, fueled by RhoA geranylgeranylation (Trebicka et al., 2007). Statin-mediated inhibition of this prenylation traps RhoA in the cytoplasm, blocking ROCK activation, myosin light chain phosphorylation and the contractile and proliferative machinery of the HSC. Schierwagen and colleagues validated this axis in the apoE−/− NASH model: simvastatin reduced hepatic inflammation and fibrosis through inhibition of RhoA and Ras, with no significant effect on steatosis or hepatic cholesterol (Schierwagen et al., 2016). In this model, in other words, the antifibrotic benefit is dissociated from the lipid effect—an observation that reinforces the isoprenoid-dependent reading. Consistent with this interpretation, Marrone and colleagues showed that both mevalonate and GGPP partially reversed the antifibrotic effects of simvastatin in activated hepatic stellate cells, underscoring the central role of isoprenoid-dependent signalling in stellate-cell biology (Marrone et al., 2015). Importantly, this observation is consistent with findings in other fibrotic diseases, where statin exposure has been associated with improved outcomes in pulmonary fibrosis, end-stage renal disease and other conditions characterized by excessive extracellular matrix deposition (Kreuter et al., 2017; Sung et al., 2022).

Layered onto this direct HSC effect is a paracrine mechanism described by the Gracia-Sancho and Bosch group. In liver sinusoidal endothelial cells (LSECs), statins induce the expression of Krüppel-like factor 2 (KLF2) in a manner dependent on inhibition of geranylgeranylation (Marrone et al., 2013). KLF2 activates vasoprotective programs in LSECs (eNOS, thrombomodulin), restores endothelial nitric oxide production and, through paracrine signaling, deactivates HSCs by reducing α-SMA, procollagen I and oxidative stress. Hepatic adenoviral overexpression of KLF2 reproduces the effect of statins, supporting the view that KLF2 acts as a mediator rather than an epiphenomenon (Marrone et al., 2015). The LSEC-KLF2-NO-HSC axis is, to our knowledge, one of the best characterized hepato-specific mechanisms at the molecular level.

The observational human correlate is the inverse association—reproducible, though constrained by the limitations of Section 2—between statin use and fibrosis progression measured by elastography. The multicentre VCTE-Prognosis study of 7,988 patients with MASLD documented attenuated progression of liver stiffness on VCTE in statin users over a median 4.6-year follow-up (Zhou et al., 2024). Schreiner and colleagues observed that statins, particularly at moderate and high intensity, are associated with reduced progression to elevated FIB-4 (Schreiner et al., 2024). Pustjens and colleagues confirmed this inverse association in advanced MASLD within a Dutch cohort, and documented that two-thirds of MASLD patients meeting contemporary criteria for statin therapy were not receiving it (Pustjens et al., 2026). The *post hoc* analysis of PROSPER in patients with FIB-4 ≥2.67 provides the only piece of indirect randomized evidence suggesting that the effect may be causal in the subgroup with non-invasively defined advanced fibrosis (de Jong et al., 2025).

Notably, the antifibrotic signal associated with statin therapy is not restricted to the liver. Consistent associations between statin exposure and improved outcomes have been reported in idiopathic pulmonary fibrosis, where *post hoc* analyses of the CAPACITY, ASCEND and INPULSIS trials, as well as population-based studies, documented slower functional decline and improved survival among statin users (Kreuter et al., 2017). Similar observations have been reported in end-stage renal disease, where statin use was associated with lower all-cause mortality independent of cardiovascular mortality (Sung et al., 2022), and in cardiovascular settings, where statin therapy reduced circulating fibrosis biomarkers and postoperative scar hypertrophy (Chang et al., 2016; Chello et al., 2021). Although these observations are similarly limited by their predominantly observational nature, their recurrence across distinct organ systems supports the concept that inhibition of mevalonate-dependent signaling pathways may exert broader antifibrotic effects. This external consistency does not prove causality in MASLD, but the recurrence of these observations across organs with distinct aetiologies yet shared fibrogenic pathways strengthens the biological plausibility that the benefits of statins in MASLD reflect modulation of fundamental mechanisms of tissue repair and fibrosis, rather than liver-specific confounding alone.

### 5.4 Hepatic carcinogenesis: the YAP/TAZ axis and the hippo pathway

The Hippo pathway and its transcriptional effectors YAP and TAZ are central regulators of hepatocyte proliferation, survival and stemness; their sustained nuclear activation is a well-established oncogenic mechanism in HCC. Sorrentino and colleagues demonstrated that YAP/TAZ are under metabolic control of the SREBP/mevalonate pathway: statin-mediated inhibition of HMG-CoA reductase reduces GGPP, which blocks the prenylation and activation of Rho GTPases and, in turn, prevents the nuclear translocation and transcriptional activity of YAP/TAZ (Sorrentino et al., 2014). Exogenous GGPP rescues YAP/TAZ activity, supporting a central role for GGPP-dependent Rho signaling in the regulation of YAP/TAZ and indicating that the effect is mediated through isoprenoid-dependent pathways rather than cholesterol itself.

Benhammou and colleagues confirmed this mechanism specifically in HCC. In human HCC cell lines (Huh7, HepG2), lipophilic statins (atorvastatin, simvastatin) induce nuclear exclusion of YAP, reduce the expression of its transcriptional targets (CTGF, CYR61), disorganize the actin cytoskeleton, and these effects are rescued specifically by GGPP rather than by cholesterol (Benhammou et al., 2023). Atorvastatin also increases the inhibitory phosphorylation of YAP at S127 and S397 and attenuates a clinical HCC risk gene signature derived from HCV-infected hepatocytes (Kim et al., 2022). The effect appears more pronounced with lipophilic than hydrophilic agents, which has both molecular plausibility (access to non-parenchymal cells) and an approximately consistent epidemiological correlate: the meta-analysis by Facciorusso and colleagues reported HR 0.49 for lipophilic statins versus 0.73 for hydrophilic statins in HCC reduction (Facciorusso et al., 2020). However, current evidence remains insufficient to establish clinical superiority of one statin class over another.

The human clinical correlate in the oncological domain demands the greatest methodological caution, for the reasons set out in Section 2: long induction period, indication bias, immortal time bias and confounding by concomitant aspirin and metformin. With those caveats, the available signal is consistent. The Zeng meta-analysis of 23 matched studies (total n ≈ 4 million) reported HR 0.52 for HCC incidence in statin users, with effect persistence after adjustment for concurrent aspirin and metformin use (Zeng et al., 2023). The Karachaliou VA cohort has added in 2026 three elements that mitigate—without eliminating—some of the classical biases: a dose-response analysis using simvastatin-equivalents as cumulative exposure, persistence of the effect after stratification by age, insulin resistance and cirrhosis status, and a specific focus on MASLD (Karachaliou et al., 2026). The Korean NHIS-Senior cohort (n = 125,926, ≥60 years) documents an analogous dose-response relationship: SHR 0.69 for users of ≥365 cDDD versus non-users, and SHR 0.63 specifically for primary liver cancer (Kang et al., 2026). Replication across independent cohorts strengthens the plausibility of causality without establishing it.

## 6 Intrahepatic hemodynamics and the decompensation-ACLF spectrum

While the four branches above rest principally on preclinical and observational evidence, intrahepatic hemodynamics is where the hepato-specific hypothesis carries the highest level of clinical validation available in this review. That validation is, however, confined to a specific niche: compensated cirrhosis with portal hypertension.

Intrahepatic vascular resistance in cirrhosis has two components: structural (fibrosis, regenerative nodules) and dynamic (sinusoidal vasoconstriction from endothelial dysfunction). The dynamic component depends on reduced intrahepatic NO bioavailability, secondary to LSEC dysfunction and increased oxidative stress. Trebicka and colleagues showed in experimental cirrhosis that statins reduce portal pressure through inhibition of RhoA/ROCK and activation of endothelial eNOS, restoring intrahepatic NO production (Trebicka et al., 2007). The human translation of this mechanism is where the evidence reaches RCT level closest to the MASLD setting. Abraldes and colleagues conducted a double-blind randomized trial in 59 cirrhotic patients with portal hypertension, showing that simvastatin 20–40 mg/day over 1 month reduces HVPG by 8.3%, improves indocyanine green clearance and adds to the effect of beta-blockers without changes in systemic hemodynamics or relevant adverse events, although the study was not powered to assess clinical outcomes such as hepatic decompensation or survival (Abraldes et al., 2009). The BLEPS trial extended this evidence: addition of simvastatin to standard therapy after variceal hemorrhage did not reduce rebleeding but was associated with improved survival (Abraldes et al., 2016). The molecular mechanism has been elucidated by the Gracia-Sancho group: induction of KLF2 in LSECs restores the vasoprotective phenotype, improves sinusoidal NO production and, through paracrine signaling, deactivates HSCs (Marrone et al., 2015; Marrone et al., 2013; Brav et al., 2019).

This hemodynamic dimension connects with the decompensation–ACLF spectrum through observational evidence that is also subject to the usual caveats. Mahmud and colleagues, in a VA cohort of 84,963 patients with cirrhosis, found an association between prior statin exposure and reduced risk of developing ACLF (HR 0.62), with an apparent dose-response relationship (Mahmud et al., 2022). Chapin and colleagues extended this finding in 11,731 patients hospitalised with grade 2–3 ACLF: prior statin exposure was associated with lower 28-day mortality (OR 0.82) and 90-day mortality (OR 0.76), again with a dose-response pattern. The effect was largest in patients with previously compensated cirrhosis (Chapin et al., 2023). Although the observational nature limits causal inference, the convergence with the experimental mechanism (KLF2 induction, HVPG reduction) makes it plausible that statins initiated in compensated cirrhosis may reduce both the incidence and the mortality of subsequent ACLF. Definitive randomized validation will likely come from the SACRED trial.

The critical qualification of the hepato-specific benefit comes from the LIVERHOPE-EFFICACY trial (Pose et al., 2025). Pose and colleagues randomized 237 patients with decompensated cirrhosis to simvastatin 20 mg plus rifaximin 1200 mg/day or placebo for 12 months; the trial was negative across all outcomes (ACLF incidence, transplantation, mortality, cirrhosis-related complications). The earlier LIVERHOPE-SAFETY trial had already shown that simvastatin 40 mg plus rifaximin increased hepatotoxicity in decompensated patients, leading to a 20 mg recommendation as the safe dose (Pose et al., 2020). The most prudent reading of the LIVERHOPE set is that the hemodynamic benefit observable in compensated cirrhosis does not extend to the decompensated stage, possibly because at that point structural damage and systemic alterations are dominant. This interpretation is consistent with the rest of the evidence: PROSPER suggests a mortality benefit in pre-cirrhotic patients with elevated FIB-4; Mahmud and Chapin report associations with reduced ACLF incidence and mortality in compensated cirrhosis with prior statin exposure; LIVERHOPE-EFFICACY confirms the absence of benefit in already-established decompensation. The composite defines, with reasonable precision, a therapeutic window situated upstream of decompensation.

## 7 Translational implications: toward a hepato-cardiovascular integration

**FIGURE 3:**
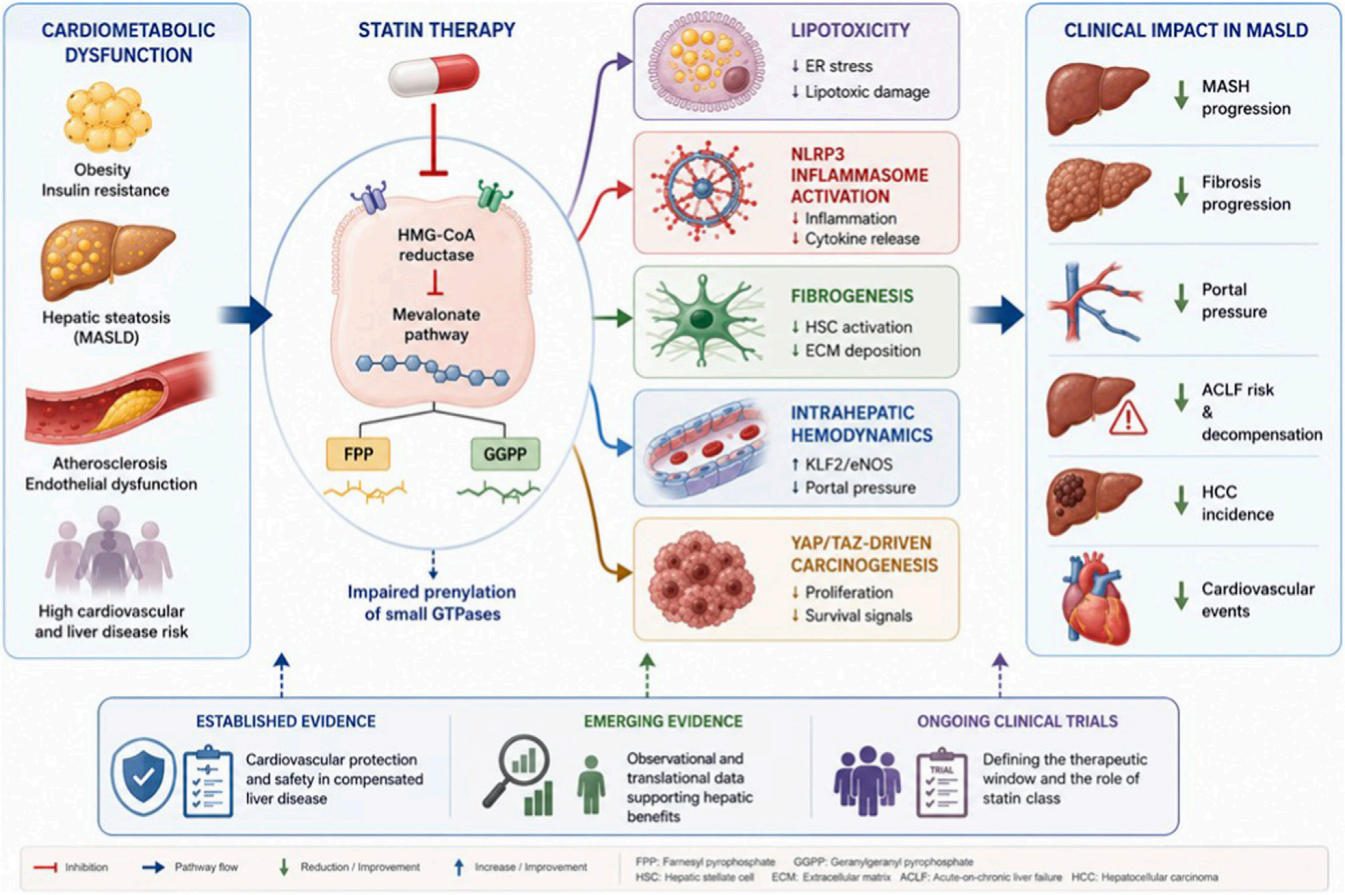
The mevalonate pathway as a shared molecular substrate for cardiovascular and hepatic protection in MASLD. This illustration presents a conceptual framework in which inhibition of the mevalonate pathway may also contribute to direct hepatic benefits. By reducing the availability of the intermediate isoprenoids farnesyl pyrophosphate (FPP) and geranylgeranyl pyrophosphate (GGPP), statins impair prenylation-dependent signaling pathways that regulate lipotoxicity, inflammasome activation, hepatic stellate cell activation, intrahepatic vascular dysfunction, and YAP/TAZ-mediated carcinogenesis. These interconnected mechanisms converge on key biological processes driving disease progression, including inflammation, fibrosis, portal hypertension, decompensation, and hepatocellular carcinoma development. Although most available evidence remains observational or translational, the remarkable consistency of the signal across independent cohorts, together with a coherent mechanistic rationale, supports the hypothesis that part of the hepatic benefit associated with statin exposure may extend beyond lipid lowering alone. Ongoing randomized clinical trials will determine whether this mechanistic framework translates into clinically meaningful liver-directed effects and define the therapeutic role of statins within the evolving treatment landscape of MASLD.

The conceptual framework proposed in this review—viewing statins in MASLD as a drug with two potentially legitimate targets, vascular and hepatic, sharing a common molecular substrate—has practical implications, as shown in Figure 3, whose strength varies with the underlying evidence. Some are reasonable hypotheses awaiting validation in ongoing trials; others are direct consequences of evidence already in hand, as mentioned in Box 1.

An immediate implication of this framework concerns the design of future trials. The most important historical gap in this literature is the absence of RCTs with primary histological endpoints in pre-cirrhotic MASLD. Three ongoing trials promise to fill it in part. STAT-NASH (NCT04679376) evaluates atorvastatin 40 mg versus placebo in statin-naïve MASH patients with F2–F3 fibrosis, with histological change as the primary endpoint (Mayo Clinic; estimated completion December 2026). SACRED (NCT03654053) is a phase 3 trial of simvastatin 40 mg versus placebo in compensated cirrhosis with portal hypertension across 11 Veterans Affairs sites, with primary clinical endpoints on decompensation, HCC and mortality (Kaplan et al., 2021). RESCU (NCT05832229) evaluates rosuvastatin 40 mg versus placebo in compensated cirrhosis within the Liver Cirrhosis Network. The results of these three trials will be decisive. The recent approval of resmetirom (Harrison et al., 2024) and semaglutide (Sanyal et al., 2025) for MASH with F2–F3 fibrosis also opens the possibility of including statins as an active arm or in combination in pragmatic trials. The characterization of KLF2, YAP/TAZ and HCC risk gene signatures potentially modulable by statins (Kim et al., 2022) suggests hepatic response biomarkers complementary to LDL.

A further implication is whether statin class influences hepatic outcomes. The lipophilic/hydrophilic distinction has a molecular basis—access to non-parenchymal cells, intensity of effect on YAP through the GGPP-Rho axis—and an approximately consistent epidemiological correlate, with a larger apparent HCC reduction for lipophilic statins (Benhammou et al., 2023; Facciorusso et al., 2020). Nevertheless, current evidence should be regarded as hypothesis-generating, and does not support preferential use of one statin class over another on the basis of hepatic outcomes alone. The dose argument is somewhat stronger: the three cohorts with dose-response analyses (Karachaliou in MASLD, Kang in older adults, Chapin in ACLF) all point to greater benefit at high intensity. There is, however, insufficient prospective randomized evidence to recommend one statin over another on the basis of the hepatic component alone, or to define an optimal dose from the hepatic perspective. Clinical choice should continue to be guided by cardiovascular risk and individual tolerance.

A third consideration relates to the rational integration of statins with newer MASH-specific therapies. Resmetirom (a selective TR-β agonist) acts hepatocentrically by accelerating β-oxidation and reducing lipotoxicity. Semaglutide acts systemically by reducing caloric pressure and inflammation, with efficacy confirmed in the most recent meta-analysis of GLP-1RA in MASH (Mantovani et al., 2025). Tirzepatide, a dual GIP/GLP-1 agonist, achieved histological MASH resolution without worsening of fibrosis in up to 62% of patients with F2–F3 MASH in the SYNERGY-NASH trial (Loomba et al., 2024). Statins, given the molecular architecture described above, would act on fibrogenesis (RhoA-KLF2 axis), inflammation (NLRP3, cholesterol crystals) and carcinogenesis (YAP). The mechanistic overlap appears limited, suggesting potential for synergy. The prespecified analysis of MAESTRO-NASH showed that resmetirom retains histological efficacy regardless of concurrent GLP-1 or SGLT2i use (Harrison et al., 2024); no signal of additive toxicity with statins has been observed. Combinations of resmetirom, statins and incretin-based agents represent a reasonable hypothesis for an integrated strategy in MASH with advanced fibrosis, but require validation in purpose-designed trials.

A final implication, probably the most clinically urgent because it does not depend on forthcoming trials, is correction of the inverse-prescription paradox. Multiple cohorts have documented that patients with more severe MASLD are precisely those who receive statins least often, presumably out of a clinically unfounded fear of hepatotoxicity (Pustjens et al., 2026; Pose et al., 2019). The paradox is doubly costly: in these patients the absolute cardiovascular benefit is maximal and, according to the evidence reviewed here with all the necessary caveats, the hepato-specific benefit may also be relevant, particularly in compensated cirrhosis. The 2024 EASL-EASD-EASO guideline has made explicit the safety of statins in MASLD even in compensated cirrhosis (Tacke et al., 2024). The Malaysian cohort of Wong and colleagues (n = 104, 12 months) confirms the absence of significant transaminase elevation across statin types and doses (Wong et al., 2025). Correcting the underprescription does not require waiting for STAT-NASH or SACRED: following the existing guideline, which has sufficient evidential basis, is enough.

Box 1 **Five questions the MASLD clinician should be able to answer today on the basis of available evidence.**

This box translates the conceptual content of the review into operational questions that arise in real consultation. It is not a substitute for individual assessment or current guidelines; it provides an interpretive map for the present moment.

**1. Are statins safe in MASLD, including compensated cirrhosis?** Yes. The 2024 EASL-EASD-EASO guideline states this explicitly. Recent observational evidence (Ayada et al., 2023; Wong et al., 2025) confirms the absence of significant transaminase elevation across statin types and doses. The historical concern about hepatotoxicity has no current evidential basis.

**2. Is there evidence that statins reduce hepatic progression in MASLD beyond their cardiovascular effect?** There is consistent, dose-dependent observational evidence, supplemented by a hypothesis-generating randomized *post hoc* analysis (PROSPER). No RCT with primary histological endpoints in pre-cirrhotic MASLD has yet reported. Three ongoing trials will provide complementary evidence: STAT-NASH is evaluating atorvastatin in MASH with histological change as the primary endpoint; SACRED is assessing simvastatin in compensated cirrhosis with clinical endpoints including hepatic decompensation, HCC and mortality; and RESCU is evaluating rosuvastatin in compensated cirrhosis, with change in liver stiffness by VCTE as the primary endpoint. Results are expected between 2026 and 2028.

**3. Should a specific statin be chosen on the basis of its hepatic component?** Not on current evidence. The choice should continue to be guided by cardiovascular risk and individual tolerance. Molecular and epidemiological grounds suggest greater hepatic potential for lipophilic statins (atorvastatin, simvastatin), but this is insufficient to support a specific prescriptive rule.

**4. Does the dose matter from the hepatic perspective?** Probably yes. Recent cohorts (Karachaliou et al., 2026; Kang et al., 2026; Chapin et al., 2023) show consistent dose-response relationships for HCC, composite hepatic events and ACLF mortality. High intensity appears more effective, within the limits of individual tolerance.

**5. Should I initiate a statin in my patient with advanced MASLD who meets cardiovascular criteria but is not receiving one?** Yes. This is the most immediate and best-supported clinical consequence of the available evidence. The documented underprescription paradox (Pustjens et al., 2026; Pose et al., 2019) is doubly costly: it forgoes the cardiovascular benefit (greatest in magnitude in these patients) and possibly also the hepato-specific benefit in the portion of the spectrum where evidence suggests statins may act.

## 8 Conclusion

The cardiovascular benefit of statins in MASLD is well established and not in question. What this review has set out to examine is whether the dominant interpretive framework should be broadened. The available clinical signal—lower hepatic mortality, attenuated fibrosis progression, lower HCC incidence, improved intrahepatic hemodynamics, lower ACLF incidence and mortality in compensated cirrhosis—is consistent in direction, partly dose-dependent and of relatively large magnitude. It remains subject, for the most part, to the intrinsic limitations of observational evidence. The *post hoc* analysis of PROSPER contributes a randomized piece consistent with this direction, although indirect with respect to MASLD.

The underlying molecular basis provides an articulated explanation: inhibition of the mevalonate pathway reduces the availability of FPP and GGPP, blocks prenylation of small GTPases and reorganises fibrogenesis, inflammation, carcinogenesis and intrahepatic hemodynamics in the organ where the statin preferentially concentrates. The clinical signal and the molecular architecture are coherent with each other.

Several limitations of this framework should be acknowledged. No RCT with primary histological endpoints in pre-cirrhotic MASLD has yet reported, though three ongoing trials (STAT-NASH, SACRED, RESCU) will produce results in the coming years. The LIVERHOPE-EFFICACY trial has shown that the benefit does not extend to decompensated cirrhosis, defining a therapeutic window upstream of consummated damage. The evidence on dose is reasonably consistent; the evidence regarding specific statins is not.

Within these limits, the conclusion this review sustains is conceptual rather than prescriptive. Viewing the statin in MASLD as a drug with two potentially legitimate targets—vascular and hepatic—that share a common molecular substrate is a reasonable hypothesis on the basis of the available evidence. It carries one immediate practical consequence that the existing guideline already supports: correcting the underprescription observed in patients with more severe MASLD. In patients with MASLD who meet cardiovascular criteria for statin prescription, statins are not a drug the liver merely tolerates. They are, at least potentially, a drug the liver uses.

## References

[B1] Abrahami D. Hudson M. Suissa S. (2021). Statins and lower mortality in rheumatic diseases: an effect of immortal time bias? Semin. Arthritis Rheum. 51, 211–218. 10.1016/j.semarthrit.2020.11.010 33385861

[B2] Abraldes J. G. Albillos A. Bañares R. Turnes J. González R. García-Pagán J. C. (2009). Simvastatin lowers portal pressure in patients with cirrhosis and portal hypertension: a randomized controlled trial. Gastroenterology 136, 1651–1658. 10.1053/j.gastro.2009.01.043 19208350

[B3] Abraldes J. G. Villanueva C. Aracil C. Turnes J. Hernandez-Guerra M. Genesca J. (2016). Addition of simvastatin to standard therapy for the prevention of variceal rebleeding does not reduce rebleeding but increases survival in patients with cirrhosis. Gastroenterology 150, 1160–1170.e3. 10.1053/j.gastro.2016.01.004 26774179

[B4] Ahmadi Y. Ghorbanihaghjo A. Argani H. (2017). The balance between induction and inhibition of mevalonate pathway regulates cancer suppression by statins: a review of molecular mechanisms. Chem. Biol. Interact. 273, 273–285. 10.1016/j.cbi.2017.06.026 28668359

[B5] Ahmadi Y. Mahmoudi N. Yousefi B. Karimian A. (2020). The effects of statins with a high hepatoselectivity rank on the extra-hepatic tissues; new functions for statins. Pharmacol. Res. 152, 104621. 10.1016/j.phrs.2019.104621 31891788

[B6] Ahsan F. Oliveri F. Goud H. K. Mehkari Z. Mohammed L. Javed M. (2020). Pleiotropic effects of statins in the light of non-alcoholic fatty liver disease and non-alcoholic steatohepatitis. Cureus 12, e10446. Published Online First. 10.7759/cureus.10446 33072455 PMC7557526

[B7] Ayada I. van Kleef L. A. Zhang H. Liu K. Li P. Abozaid Y. J. (2023). Dissecting the multifaceted impact of statin use on fatty liver disease: a multidimensional study. EBioMedicine 87, 104392. 10.1016/j.ebiom.2022.104392 36502575 PMC9758527

[B8] Benhammou J. N. Qiao B. Ko A. Sinnett-Smith J. Pisegna J. R. Rozengurt E. (2023). Lipophilic statins inhibit YAP coactivator transcriptional activity in HCC cells through rho-mediated modulation of actin cytoskeleton. Am. J. Physiology-Gastrointestinal Liver Physiology 325, G239–G250. 10.1152/ajpgi.00089.2023 37366601 PMC10511177

[B9] Bravo M. Raurell I. Hide D. Fernández-Iglesias A. Gil M. Barberá A. (2019). Restoration of liver sinusoidal cell phenotypes by statins improves portal hypertension and histology in rats with NASH. Sci. Rep. 9, 20183. 10.1038/s41598-019-56366-2 31882668 PMC6934751

[B10] Brookhart M. A. Patrick A. R. Dormuth C. Avorn J. Shrank W. Cadarette S. M. (2007). Adherence to lipid-lowering therapy and the use of preventive health services: an investigation of the healthy user effect. Am. J. Epidemiol. 166, 348–354. 10.1093/aje/kwm070 17504779

[B11] Chang Y.-Y. Wu Y.-W. Lee J.-K. Lin Y. M. Lin Y. T. Kao H. L. (2016). Effects of 12 weeks of atorvastatin therapy on myocardial fibrosis and circulating fibrosis biomarkers in Statin-NaïVe patients with hypertension with atherosclerosis. J. Investigative Med. 64, 1194–1199. 10.1136/jim-2016-000092 27430242

[B12] Chapin S. Kaplan D. E. Taddei T. Mahmud N. (2023). Association between statin exposure and short-term mortality in patients with high-grade acute-on-chronic liver failure. JHEP Rep. 5, 100740. 10.1016/j.jhepr.2023.100740 37215188 PMC10193237

[B13] Chello C. Nenna A. Chello M. Satriano U. M. Cardetta F. Lusini M. (2021). Statin treatment and hypertrophic scarring after cardiac surgery. Wound Repair Regen. 29, 129–133. 10.1111/wrr.12878 33236817

[B14] Climent E. Benaiges D. Pedro-Botet J. (2021). Hydrophilic or lipophilic statins? Front. Cardiovasc Med. 8, 687585. 10.3389/fcvm.2021.687585 34095267 PMC8172607

[B15] Commins I. Clayton-Chubb D. Janko N. Majeed A. Kemp W. Roberts S. K. (2026). Efficacy and safety of statins in MASLD and other chronic liver diseases. Med. Sci. 14, 84. 10.3390/medsci14010084 41718130 PMC12922148

[B16] de Jong V. Theel W. Castro C. M. Grobbee D. E. Jukema W. Trompet S. (2025). Pravastatin reduces all-cause mortality in elderly individuals at risk of liver fibrosis: post hoc analysis of the PROSPER trial. JHEP Rep. 7, 101337. 10.1016/j.jhepr.2025.101337 40212790 PMC11985108

[B17] Facciorusso A. Abd El Aziz M. A. Singh S. Pusceddu S. Milione M. Giacomelli L. (2020). Statin use decreases the incidence of hepatocellular carcinoma: an updated meta-analysis. Cancers (Basel) 12, 874. 10.3390/cancers12040874 32260179 PMC7225931

[B18] Harrison S. A. Bedossa P. Guy C. D. Schattenberg J. M. Loomba R. Taub R. (2024). A phase 3, randomized, controlled trial of resmetirom in NASH with liver fibrosis. N. Engl. J. Med. 390, 497–509. 10.1056/nejmoa2309000 38324483

[B19] Hirsova P. Ibrahim S. H. Krishnan A. Verma V. K. Bronk S. F. Werneburg N. W. (2016). Lipid-induced signaling causes release of inflammatory extracellular vesicles from hepatocytes. Gastroenterology 150, 956–967. 10.1053/j.gastro.2015.12.037 26764184 PMC4808464

[B20] Inia J. A. Stokman G. Pieterman E. J. Morrison M. C. Menke A. L. Verschuren L. (2023). Atorvastatin attenuates diet-induced non-alcoholic steatohepatitis in APOE*3-Leiden mice by reducing hepatic inflammation. Int. J. Mol. Sci. 24, 7818. 10.3390/ijms24097818 37175538 PMC10178767

[B21] Ioannou G. N. Haigh W. G. Thorning D. Savard C. (2013). Hepatic cholesterol crystals and crown-like structures distinguish NASH from simple steatosis. J. Lipid Res. 54, 1326–1334. 10.1194/jlr.M034876 23417738 PMC3622327

[B22] Ioannou G. N. Subramanian S. Chait A. Haigh W. G. Yeh M. M. Farrell G. C. (2017). Cholesterol crystallization within hepatocyte lipid droplets and its role in murine NASH. J. Lipid Res. 58, 1067–1079. 10.1194/jlr.M072454 28404639 PMC5456359

[B23] Ioannou G. N. Horn C. L. Kothari V. Yeh M. M. Shyu I. Lee S. P. (2023). Genetic deletion or pharmacologic inhibition of the Nlrp3 inflammasome did not ameliorate experimental NASH. J. Lipid Res. 64, 100330. 10.1016/j.jlr.2023.100330 36641116 PMC9944495

[B24] Kakazu E. Mauer A. S. Yin M. Malhi H. (2016). Hepatocytes release ceramide-enriched pro-inflammatory extracellular vesicles in an IRE1α-dependent manner. J. Lipid Res. 57, 233–245. 10.1194/jlr.M063412 26621917 PMC4727419

[B25] Kang E. S. Kim H. J. Park S. J. Kang J. H. Park S. Sha M. (2026). Statin use and the risk of liver-related events in older adults with steatotic liver disease. Sci. Rep. 16, 12615. 10.1038/s41598-026-43347-5 41796248 PMC13087278

[B26] Kaplan D. E. Mehta R. Garcia-Tsao G. Albrecht J. Aytaman A. Baffy G. (2021). SACRED: effect of simvastatin on hepatic decompensation and death in subjects with high-risk compensated cirrhosis: statins and cirrhosis: reducing events of decompensation. Contemp. Clin. Trials 104, 106367. 10.1016/j.cct.2021.106367 33771685 PMC8422958

[B27] Karachaliou G. S. Perkins A. M. Dorn C. Reeves R. M. Arnold T. Bashir M. R. (2026). Association of statin use with reduced primary liver cancer risk, independent of age and cirrhosis protection in MASLD. Cancers (Basel) 18, 1132. 10.3390/cancers18071132 41976355 PMC13072028

[B28] Kim K.-Y. Jang H.-J. Yang Y. R. Park K. I. Seo J. Shin I. W. (2016). SREBP-2/PNPLA8 axis improves non-alcoholic fatty liver disease through activation of autophagy. Sci. Rep. 6, 35732. 10.1038/srep35732 27767079 PMC5073315

[B29] Kim M. Kim M. Salloum S. Qian T. Wong L. P. Xu M. (2022). Atorvastatin favorably modulates a clinical hepatocellular carcinoma risk gene signature. Hepatol. Commun. 6, 2581–2593. 10.1002/hep4.1991 35712812 PMC9426409

[B30] Kreuter M. Bonella F. Maher T. M. Costabel U. Spagnolo P. Weycker D. (2017). Effect of statins on disease-related outcomes in patients with idiopathic pulmonary fibrosis. Thorax 72, 148–153. 10.1136/thoraxjnl-2016-208819 27708114 PMC5284334

[B31] Ktenopoulos N. Sagris M. Gerogianni M. Pamporis K. Apostolos A. Balampanis K. (2024). Non-alcoholic fatty liver disease and coronary artery disease: a bidirectional association based on endothelial dysfunction. Int. J. Mol. Sci. 25, 10595. 10.3390/ijms251910595 39408924 PMC11477211

[B32] Lee L.-T. Huang H.-Y. Huang K.-C. Chen C. Y. Lee W. C. (2009). Age-period-cohort analysis of hepatocellular carcinoma mortality in Taiwan, 1976–2005. Ann. Epidemiol. 19, 323–328. 10.1016/j.annepidem.2008.12.013 19362276

[B33] Levesque L. E. Hanley J. A. Kezouh A. Suissa S. (2010). Problem of immortal time bias in cohort studies: example using statins for preventing progression of diabetes. BMJ 340, b5087. 10.1136/bmj.b5087 20228141

[B34] Loomba R. Hartman M. L. Lawitz E. J. Vuppalanchi R. Boursier J. Bugianesi E. (2024). Tirzepatide for metabolic dysfunction–associated steatohepatitis with liver fibrosis. N. Engl. J. Med. 391, 299–310. 10.1056/NEJMoa2401943 38856224

[B35] Mahmud N. Chapin S. Goldberg D. S. Reddy K. R. Taddei T. H. Kaplan D. E. (2022). Statin exposure is associated with reduced development of acute-on-chronic liver failure in a veterans affairs cohort. J. Hepatol. 76, 1100–1108. 10.1016/j.jhep.2021.12.034 35066085 PMC9018495

[B36] Mantovani A. Morandin R. Fiorio V. Lando M. G. Stefan N. Tilg H. (2025). Glucagon‐like Peptide‐1 receptor agonists improve MASH and liver fibrosis: a meta‐analysis of randomised controlled trials. Liver Int. 45, e70256. 10.1111/liv.70256 40736113 PMC12309150

[B37] Marrone G. Russo L. Rosado E. Hide D. García-Cardeña G. García-Pagán J. C. (2013). The transcription factor KLF2 mediates hepatic endothelial protection and paracrine endothelial–stellate cell deactivation induced by statins. J. Hepatol. 58, 98–103. 10.1016/j.jhep.2012.08.026 22989565

[B38] Marrone G. Maeso-Díaz R. García-Cardena G. Abraldes J. G. García-Pagán J. C. Bosch J. (2015). KLF2 exerts antifibrotic and vasoprotective effects in cirrhotic rat livers: behind the molecular mechanisms of statins. Gut 64, 1434–1443. 10.1136/gutjnl-2014-308338 25500203

[B39] McTaggart S. J. (2006). Isoprenylated proteins. Cell Mol. Life Sci. 63, 255–267. 10.1007/s00018-005-5298-6 16378247 PMC11136304

[B40] Mridha A. R. Wree A. Robertson A. A. B. Yeh M. M. Johnson C. D. Van Rooyen D. M. (2017). NLRP3 inflammasome blockade reduces liver inflammation and fibrosis in experimental NASH in mice. J. Hepatol. 66, 1037–1046. 10.1016/j.jhep.2017.01.022 28167322 PMC6536116

[B41] Oesterle A. Laufs U. Liao J. K. (2017). Pleiotropic effects of statins on the cardiovascular system. Circ. Res. 120, 229–243. 10.1161/CIRCRESAHA.116.308537 28057795 PMC5467317

[B42] Palatini P. De Martin S. (2016). Pharmacokinetic drug interactions in liver disease: an update. World J. Gastroenterol. 22, 1260–1278. 10.3748/wjg.v22.i3.1260 26811663 PMC4716036

[B43] Patrick A. R. Shrank W. H. Glynn R. J. Solomon D. H. Dormuth C. R. Avorn J. (2011). The association between statin use and outcomes potentially attributable to an unhealthy lifestyle in older adults. Value Health 14, 513–520. 10.1016/j.jval.2010.10.033 21669377 PMC5059150

[B44] Pose E. Trebicka J. Mookerjee R. P. Angeli P. Ginès P. (2019). Statins: old drugs as new therapy for liver diseases? J. Hepatol. 70, 194–202. 10.1016/j.jhep.2018.07.019 30075229

[B45] Pose E. Napoleone L. Amin A. Campion D. Jimenez C. Piano S. (2020). Safety of two different doses of simvastatin plus rifaximin in decompensated cirrhosis (LIVERHOPE-SAFETY): a randomised, double-blind, placebo-controlled, phase 2 trial. Lancet Gastroenterol. Hepatol. 5, 31–41. 10.1016/S2468-1253(19)30320-6 31607677

[B46] Pose E. Jiménez C. Zaccherini G. Campion D. Piano S. Uschner F. E. (2025). Simvastatin and rifaximin in decompensated cirrhosis. JAMA 333, 864. 10.1001/jama.2024.27441 39908052 PMC11800124

[B47] Pustjens J. van Kleef L. A. Pavlović J. Lahousse L. Holleboom A. G. Janssen H. L. A. (2026). Underuse of statins in MASLD despite population-based associations with lower liver stiffness. JHEP Rep. 8, 101764. 10.1016/j.jhepr.2026.101764 41861677 PMC13019549

[B48] Rinella M. E. Lazarus J. V. Ratziu V. Francque S. M. Sanyal A. J. Kanwal F. (2023). A multisociety Delphi consensus statement on new fatty liver disease nomenclature. J. Hepatol. 79, 1542–1556. 10.1016/j.jhep.2023.06.003 37364790

[B49] Sanyal A. J. Newsome P. N. Kliers I. Østergaard L. H. Long M. T. Kjær M. S. (2025). Phase 3 trial of semaglutide in metabolic dysfunction–associated steatohepatitis. N. Engl. J. Med. 392, 2089–2099. 10.1056/NEJMoa2413258 40305708

[B50] Schachter M. (2005). Chemical, pharmacokinetic and pharmacodynamic properties of statins: an update. Fundam. Clin. Pharmacol. 19, 117–125. 10.1111/j.1472-8206.2004.00299.x 15660968

[B51] Schierwagen R. Maybüchen L. Hittatiya K. Klein S. Uschner F. E. Braga T. T. (2016). Statins improve NASH via inhibition of RhoA and ras. Am. J. Physiology-Gastrointestinal Liver Physiology 311, G724–G733. 10.1152/ajpgi.00063.2016 27634010

[B52] Schreiner A. D. Zhang J. Petz C. A. Moran W. P. Koch D. G. Marsden J. (2024). Statin prescriptions and progression of advanced fibrosis risk in primary care patients with MASLD. BMJ Open Gastroenterol. 11, e001404. 10.1136/bmjgast-2024-001404 39019623 PMC11256061

[B53] Shrank W. H. Patrick A. R. Alan Brookhart M. (2011). Healthy user and related biases in observational studies of preventive interventions: a primer for physicians. J. Gen. Intern Med. 26, 546–550. 10.1007/s11606-010-1609-1 21203857 PMC3077477

[B54] Sorrentino G. Ruggeri N. Specchia V. Cordenonsi M. Mano M. Dupont S. (2014). Metabolic control of YAP and TAZ by the mevalonate pathway. Nat. Cell Biol. 16, 357–366. 10.1038/ncb2936 24658687

[B55] Sung F.-C. Jong Y.-C. Muo C.-H. Hsu C. C. Tsai W. C. Hsu Y. H. (2022). Statin therapy for hyperlipidemic patients with chronic kidney disease and end-stage renal disease: a retrospective cohort study based on 925,418 adults in Taiwan. Front. Pharmacol. 13, 815882. 10.3389/fphar.2022.815882 35308209 PMC8930832

[B56] Tacke F. Horn P. Wong V. W. S. Ratziu V. Bugianesi E. Francque S. (2024). EASL–EASD–EASO clinical practice guidelines on the management of metabolic dysfunction-associated steatotic liver disease (MASLD). J. Hepatol. 81, 492–542. 10.1016/j.jhep.2024.04.031 38851997

[B57] Targher G. Byrne C. D. Tilg H. (2020). NAFLD and increased risk of cardiovascular disease: clinical associations, pathophysiological mechanisms and pharmacological implications. Gut 69, 1691–1705. 10.1136/gutjnl-2020-320622 32321858

[B58] Trebicka J. Hennenberg M. Laleman W. Shelest N. Biecker E. Schepke M. (2007). Atorvastatin lowers portal pressure in cirrhotic rats by inhibition of RhoA/rho-kinase and activation of endothelial nitric oxide synthase. Hepatology 46, 242–253. 10.1002/hep.21673 17596891

[B59] Wong P. J. W. Ng S. C. George S. Rasdi R. B. (2025). Impact of statins on liver transaminases in patients with metabolic dysfunction–associated steatotic liver disease: a 12-month retrospective review. Hepatol. Forum, 7 (1), 21–25. 10.14744/hf.2025.02335 41589203 PMC12831980

[B60] Xu W. Lee A. L. Lam C. L. K. Danaei G. Wan E. Y. F. (2024). Benefits and risks associated with statin therapy for primary prevention in old and very old adults. Ann. Intern Med. 177, 701–710. 10.7326/M24-0004 38801776

[B61] Yun B. Park H. Lee J. Kim B. K. Yoon J. H. (2025). Statin use and liver-related prognosis among patients with MASLD. JHEP Rep. 7, 101313. 10.1016/j.jhepr.2024.101313 40124167 PMC11929059

[B62] Zeng R. W. Yong J. N. Tan D. J. H. Fu C. E. Lim W. H. Xiao J. (2023). Meta‐analysis: chemoprevention of hepatocellular carcinoma with statins, aspirin and metformin. Aliment. Pharmacol. Ther. 57, 600–609. 10.1111/apt.17371 36625733 PMC10792521

[B63] Zhou X.-D. Kim S. U. Yip T. C.-F. Petta S. Nakajima A. Tsochatzis E. (2024). Long-term liver-related outcomes and liver stiffness progression of statin usage in steatotic liver disease. Gut 73, 1883–1892. 10.1136/gutjnl-2024-333074 39089860

